# Dietary Patterns, Cardiorespiratory and Muscular Fitness in 9–11-Year-Old Children from Dunedin, New Zealand

**DOI:** 10.3390/nu10070887

**Published:** 2018-07-10

**Authors:** Pouya Saeedi, Katherine E. Black, Jillian J. Haszard, Sheila Skeaff, Lee Stoner, Brittany Davidson, Harriet A. L. Harrex, Kim Meredith-Jones, Robin Quigg, Jyh Eiin Wong, Paula M. L. Skidmore

**Affiliations:** 1 Department of Human Nutrition, University of Otago, Dunedin 9054, New Zealand; pouya.saeedi@gmail.com (P.S.); katherine.black@otago.ac.nz (K.E.B.); jill.haszard@otago.ac.nz (J.J.H.); sheila.skeaff@otago.ac.nz (S.S.); brittany_davison@hotmail.com (B.D.); harriet.harrex@outlook.com (H.A.L.H.); 2 Department of Exercise and Sports Science, University of North Carolina, Chapel Hill, NC 27519, USA; stonerl@email.unc.edu; 3 Department of Medicine, University of Otago, Dunedin 9054, New Zealand; kim.meredith-jones@otago.ac.nz; 4 Cancer Society Social and Behavioural Research Unit, Department of Preventive and Social Medicine, Dunedin School of Medicine, University of Otago, Dunedin 9054, New Zealand; robin.quigg@otago.ac.nz; 5 Centre for Community Health, Faculty of Health Sciences, Universiti Kebangsaan Malaysia, Kuala Lumpur 50300, Malaysia; wjeiin@ukm.edu.my

**Keywords:** $\dot{V}$O_2max_, handgrip strength, data-driven dietary patterns, children, New Zealand

## Abstract

Research shows that cardiorespiratory (CRF) and muscular fitness in childhood are associated with a healthier cardiovascular profile in adulthood. Identifying factors associated with measures of fitness in childhood could allow for strategies to optimize cardiovascular health throughout the lifecourse. The aim of this study was to examine the association between dietary patterns and both CRF and muscular fitness in 9–11-year-olds. In this study of 398 children, CRF and muscular fitness were assessed using a 20-m shuttle run test and digital hand dynamometer, respectively. Dietary patterns were derived using principal component analysis. Mixed effects linear regression models were used to assess associations between dietary patterns and CRF and muscular fitness. Most children had healthy CRF (99%, FITNESSGRAM) and mean ± SD muscular fitness was 15.2 ± 3.3 kg. Two dietary patterns were identified; “Snacks” and “Fruit and Vegetables”. There were no significant associations between either of the dietary patterns and CRF. Statistically significant but not clinically meaningful associations were seen between dietary patterns and muscular fitness. In an almost exclusively fit cohort, food choice is not meaningfully related to measures of fitness. Further research to investigate diet-fitness relationships in children with lower fitness levels can identify key populations for potential investments in health-promoting behaviors.

## 1. Introduction

Cardiovascular diseases (CVD) remain the leading cause of mortality worldwide [1]. Although CVD generally manifests in adulthood, evidence has increasingly shown its underlying origins in childhood [2]. From a young age, lifestyle-related factors are shown to have an important role in minimizing or even preventing CVD and underlying risk factors [3,4,5], including cardiorespiratory (CRF) and muscular fitness [6,7]. Low levels of CRF and muscular fitness are associated with CVD risk factors such as adiposity, clustering of metabolic risk factors, and C-reactive protein in childhood and early adolescence [4,5]. A healthy diet, characterized by high consumption of foods such as leafy greens, fruit, poultry, fish, and dairy products, as an important component of an overall healthy lifestyle, is inversely associated with the same CVD risk factors, including adiposity in childhood [3] and may prevent the development of CVD later in life. Therefore, identification of healthier dietary factors associated with CRF and muscular fitness in children could not only provide strategies to improve these measures of fitness but also provide an opportunity to possibly maintain healthy levels of those classic CVD risk factors.

However, there is insufficient information on the association between physical fitness and dietary habits in children. A limited number of studies have examined relationships between dietary patterns and CRF [6,8,9,10] and muscular fitness [6,9,11]. In this previous research, healthier dietary patterns were found to be associated with both measures of fitness. The premise of these results is that the components of healthier diet, including high levels of antioxidants would theoretically lead to increase efficiency in oxygen uptake and utilization, as well as protection of cellular components such as proteins from the catabolic effects of oxidative stress that is the imbalance between the production of reactive oxygen species and antioxidant defense [12].

However, the majority of these studies were conducted in adolescents, rather than younger children. In adolescents, the relationships may differ based on changes in hormone levels during puberty that may affect CRF and muscle mass, or due to less parental control over the diet [13,14]. It is, therefore, essential to identify what aspects of children’s dietary habits are associated with physical fitness, in order to minimize undesirable and optimize positive behaviors affecting children’s current health and therefore reduce the risk of developing CVD later in life. Therefore, the aim of this study was to examine associations between dietary patterns and both CRF and muscular fitness in a sample of 9–11-year-old children.

## 2. Materials and Methods

### 2.1. Study Design and Participants

**Figure 1:**
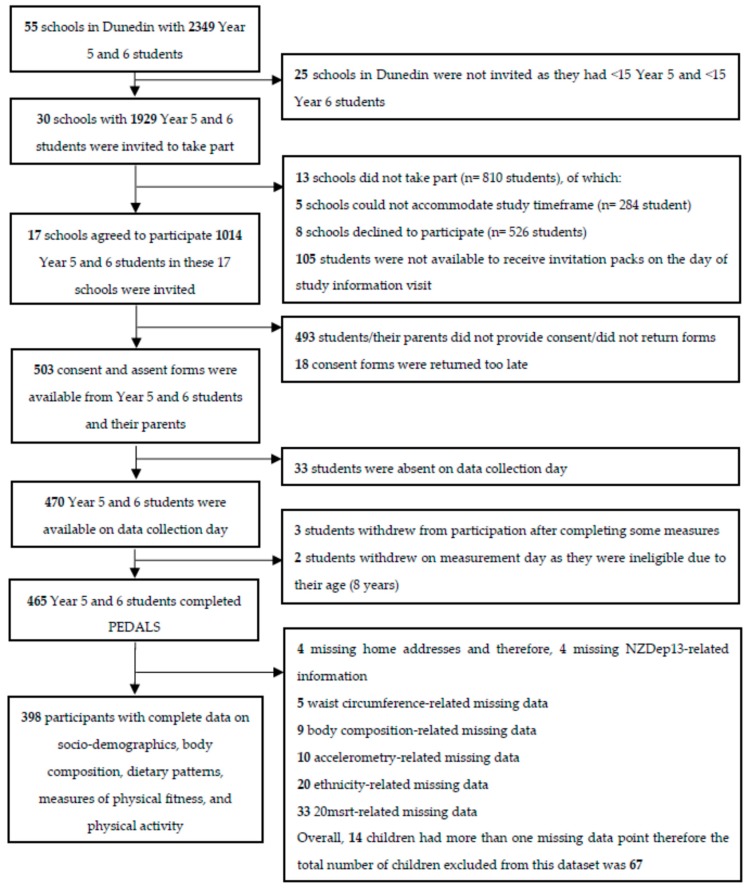
Flowchart illustrating the selection of schools and children into the Physical activity, Exercise, Diet And Lifestyle Study (PEDALS). NZDep13, New Zealand deprivation index.

The current observational study used data collected as part of the Physical activity, Exercise, Diet, And Lifestyle Study (PEDALS). PEDALS was an epidemiological study, conducted in primary schools in Dunedin, New Zealand, between April and December 2015. All primary schools in Dunedin with more than 15 Year 5 and 6 students (9–11 years old) were invited to take part in PEDALS via e-mail/letter invitation to the school principal. If schools agreed to participate, all Year 5 and 6 students in those schools were invited to take part (Figure 1). Students who were interested were given invitation packs, which included a parental information sheet and consent form, the child’s information sheet and assent form, a physical activity readiness questionnaire (PAR-Q) to be completed by a parent/primary caregiver on behalf of the child, and a pre-paid postage envelope. The PAR-Q was used to screen for eligibility to participate in the CRF test. Participants were excluded if they had any of the following conditions: chest pain caused by physical activity; epilepsy; dizziness or fainting; a bone, joint or muscular problem; and/or if their doctor had ever advised them not to exercise. Both the parent’s consent and child’s assent were necessary for participating in the study. Participants were free to withdraw consent and to discontinue participation in the study at any time without prejudice to the subject.

Data collection included completion of a self-reported food frequency questionnaire (PEDALS-FFQ) and physical measurements (physical fitness, physical activity, and anthropometric measurements). Comprehensive training sessions were performed prior to data collection to ensure all study researchers were familiar with the measurement protocols and procedures. Data collection was conducted at the participant’s school during school hours (approximately between 9 a.m. to 3 p.m.). The ethics committee of the University of Otago approved the study (Ref No. 14/227), in accordance with the declaration of Helsinki (2004).

### 2.2. Study Measurements

#### Cardiorespiratory Fitness (CRF)

CRF of participants was determined as maximal oxygen uptake ($\dot{V}$O_2max_), obtained from a 20-m shuttle run test (20msrt). Participants were asked to run continuously between two lines 20 meters apart (usually in groups of 12–15, depending on the number of participants at each school), following a pre-recorded signal. The running speed started at eight km·h^−1^ in the first level, increasing by 0.5 km·h^−1^ every minute (each level is equal to one minute), except the first level where the speed increased by one km·h^−1^ (in line with the EUROFIT protocol). If participants reached the line before the signal, they were asked to wait for the signal before continuing to run again. Furthermore, if participants reached the line after the signal, they were given a warning to continue running so they could catch up. If they failed to reach the line before the signal on two consecutive occasions, they were asked to stop running and were withdrawn from the test. Children received verbal encouragement to push themselves while running. The study researcher then recorded the participant’s last completed level and stage.

There are several equations available to estimate $\dot{V}$O_2max_ from 20msrt. Of the validated equations used in children [15,16], we chose the Léger equation, (1) to predict $\dot{V}$O_2max_ values, as our population more closely resembled the population in which the Léger equation had been validated than those from other studies. The Léger equation predicts $\dot{V}$O_2max_ taking into account the maximal aerobic shuttle run speed (X_1_) and participant’s age (X_2_) [15]:

(1) $$\dot{V}O_{2\max}=(\mathrm{mL}\cdot {\mathrm{kg}}^{-1}\cdot \min^{-1})=31.025+3.238X_{1}-3.248X_{2}+0.1536X_{1}X_{2}$$

The value of X_1_ was obtained based on the last stage completed during the 20msrt and this was matched with speed (km·h^−1^) from the EUROFIT standard chart. Predicted $\dot{V}$O_2max_ using the Léger equation is a valid (*r* = 0.71) and reproducible (*r* = 0.89) measure in children [15]. The FITNESSGRAM cut-offs were used to categorize participants based on their $\dot{V}$O_2max_ values into the following groups: needs improvement-health risk zone ($\dot{V}$O_2max_ ≤ 37.3 mL·kg^−1^.min^−1^), needs improvement (37.4 ≤ $\dot{V}$O_2max_ ≤ 40.1 mL·kg^−1^·min^−1^), and healthy fitness zones ($\dot{V}$O_2max_ ≥ 40.2 mL·kg^−1^·min^−1^) [17].

### 2.3. Muscular Fitness

A digital hand dynamometer (Camry, EH101, Zhongshan Camry Electronic Co. Ltd., Zhongshan, China) was used to measure handgrip strength of both dominant and non-dominant hands, as a measure of muscular fitness. Handgrip dynamometry is the most commonly used test to measure upper body muscular fitness as it is inexpensive, time efficient, easy to perform, and well-tolerated by children. The validity (moderate-strong), reliability (strong), and feasibility of handgrip dynamometry for use in children irrespective of age and sex has been documented [18,19].

Participants were asked to sit in a chair with their feet touching the floor, shoulders neutrally rotated, elbow flexed to 90 degrees, and forearm in a neutral position, based on the proposed protocol from the American Society of Hand Therapists. The grip size of the dynamometer was adjusted to the participant’s hand size, if needed. After familiarizing children with the dynamometer, on the count of three, participants were asked to exert a maximal force on the dynamometer, squeezing as hard as they could for three seconds. Instruction and verbal encouragement during muscular fitness assessment (e.g., how long an individual squeezes the dynamometer for) may affect an individual’s performance in the test. Therefore, using a standardized protocol to measure muscular fitness is advised. Three trials were performed for each hand, alternating between dominant and non-dominant hands. The mean value of the three trials in both dominant and non-dominant hands was used in data analyses.

#### 2.3.1. Dietary Intake

A validated non-quantitative food frequency questionnaire (FFQ), the PEDALS-FFQ, was used to assess frequency of consumption of 28 food items/groups during a usual week including; fruit, vegetables, yoghurt, cheese, standard milk (full fat milk; blue top), trim milk (low fat/skimmed milk; green top), breakfast cereals, bread (white, wholemeal), potato, rice, pasta, meat (processed, others), fish, non-dairy drinks (fruit juice, diet/standard fizzy drinks), sandwich spreads, tomato sauce, ketchup, salty and sweet snacks, and sweet bakery. The relative validity and reproducibility of the PEDALS-FFQ has been previously examined in a sample of primary school-aged children in Dunedin, New Zealand [20].

#### 2.3.2. Assessment of Covariates

Participants were prioritized into three ethnic groups, according to the methods used in national surveys in New Zealand; Māori (indigenous population of New Zealand), Pacific people (e.g., Samoan, Cook Island Maori, Niuean), and New Zealand European and other ethnicities (e.g., Indian and Chinese) [21]. For the purpose of data analysis children were categorized into two groups: Māori and non-Māori. As there were a small number of Pacific people in this study (*n* = 10) they were also placed in the non-Māori group. The New Zealand deprivation index (NZDep13) was used to categorize participants based on their socio-economic status; low deprivation (NZDep13 scores between 1–3), middle deprivation (NZDep13 scores between 4–7), and high deprivation (NZDep13 scores between 8–10) [22].

A portable stadiometer (WSHRP, Wedderburn^®^, New Zealand) was used to measure children’s height to the nearest 0.1 cm, with their head in the Frankfort Plane position. If the first two readings differed by more than 0.5 cm, a third measurement was obtained. Children’s weight and body composition was measured once, using a foot-to-foot bioelectrical impedance analysis (BIA) machine (TBF-300A, Tanita, Japan). Children’s fat mass index (FMI) and fat-free mass index (FFMI) were calculated using information collected from the BIA. FMI (kg·m^−2^) was calculated as fat mass (kg) divided by height in meters squared (m^2^). Furthermore, FFMI (kg·m^−2^) was determined by dividing fat-free mass (kg) by height in meters squared (m^2^). BMI (kg·m^−2^) was calculated from measured height and weight by dividing weight (kg) to height as meters squared (m^2^). To assess children’s weight status, the International Obesity Task Force (IOTF) BMI cut-offs were used to group children into four categories as follows; underweight, normal weight, overweight, and obese [23]. As there were only a small number of underweight participants (*n* = 21), they were grouped with their normal weight counterparts (‘underweight-normal weight’ group), while the overweight and obese participants were also grouped together (‘overweight-obese’ group). BMI-for-age z-scores were calculated using the World Health Organization (WHO) method.

A wrist-worn accelerometer (Actigraph GT3X+, Pensacola, FL, USA) was used to objectively measure physical activity. Participants wore the accelerometer on their non-dominant wrist for eight consecutive days at all times, except when showering or playing water sports. Accelerometers were initialized with a 30 Hz sampling rate and 5-s epoch. Accelerometers measure activity in raw counts that are then processed to determine overall activity patterns and activity at different intensities. An automated MATLAB-based script was used to clean and score accelerometer data and remove sleep periods prior to activity analyses. At least eight hours of wear time was considered as a valid day and if participants had less than three valid days of wearing the accelerometer they were excluded from the analysis. Twenty consecutive zero counts, during awake periods only, was considered as non-wear time [24]. The minimum wear time of three days for more than eight hours has been shown to produce reproducible (intraclass correlation coefficient: ICC > 0.7) estimates of habitual physical activity in children [25]. To determine moderate-vigorous physical activity (hours per day) of children, Chandler et al.’s cut-points per five-second epochs, developed for wrist-worn accelerometers in 8–12 year-old children were used [26].

### 2.4. Sample Size

The PEDALS study was designed to primarily determine the prevalence of food choices, physical activity behaviors, and fitness in Dunedin children aged 9–11 years old. Secondary objectives were to assess relationships between parent and child measures (such as diet and psychological well-being) and between predictors of health such as diet and arterial stiffness in children. Using clustered sampling with schools as the sampling unit, a design effect of three was used [27]. To determine prevalence to a precision of at least ±9%, 357 participants would need to be recruited. To allow for 10% incomplete data it was decided to aim for recruitment of at least 400 children and their parent(s). This sample size would also be sufficient to assess the secondary objectives using appropriate regression models, which are unlikely to be affected by clustering effects.

### 2.5. Statistical Analyses

Stata 12.1 (StataCorp, College Station, TX, USA) was used for all statistical analyses. Dietary patterns were identified from the PEDALS-FFQ data using the principal component analysis (PCA) approach with varimax orthogonal rotation. Detailed information on the dietary pattern derivation has been reported elsewhere [28]. A scree plot was used to identify the number of dietary patterns produced. Dietary pattern scores (factor loading multiplied by the frequency of food consumption) were generated and standardized so that each child had a score for each of the identified dietary patterns. Missing data from the PEDALS-FFQ were imputed with a worst-case scenario response only if at least 75% of the FFQ questions had been completed.

Bivariable analyses examining the relationship between demographic, anthropometric, and lifestyle predictors (i.e., age, sex, ethnicity, NZDep13, BMI z-score, BMI categories, FMI, FFMI, moderate-vigorous physical activity, and dietary pattern scores) and components of physical fitness (i.e., CRF and muscular fitness) were investigated using mixed effects regression models, with school as a random effect (clusters). Beta coefficients are presented for continuous variables representing the mean difference in $\dot{V}$O_2max_ (mL·kg^−1^·min^−1^) or muscular fitness (kg) associated with each unit increase in the predictor. Mean and standard deviation (SD) for each category are presented for categorical predictors.

To assess the independent association between dietary patterns and CRF ($\dot{V}$O_2max_) and muscular fitness, mixed effects regression models with robust standard errors and school as a random effect were used. Two separate regression models were generated, adjusted for known covariates based on the available literature and findings from the bivariable models. The first model assessed the relationship between dietary patterns and components of physical fitness (i.e., CRF or muscular fitness) with adjustment for age and sex (Model 1). As ethnicity [29], socio-economic status [29,30], FMI [31,32,33], FFMI [33], height [32], and moderate-vigorous physical activity [7,31] have been found to be associated with CRF, these factors were further included in Model 2 that assessed the relationship between dietary patterns and CRF. For the investigation of the relationship between dietary patterns and muscular fitness, Model 2 included covariates from Model 1, as well as ethnicity [34], NZDep13 [30], FFMI [35], height [35], and moderate-vigorous physical activity [36].

Regression models were further checked for the moderation effects of sex and weight status on the association between dietary pattern scores and components of physical fitness (i.e., CRF and muscular fitness) by the inclusion of an interaction term between sex/weight status and dietary pattern score. If there was no indication of a “sex-dietary pattern score” or “weight status-dietary pattern score” interaction, the interaction term was removed from the model and results were presented without stratification by sex or weight status, otherwise stratified analyses would be presented

## 3. Results

### 3.1. Characteristics of Participants

Of 30 eligible schools, 17 agreed to participate in PEDALS (56.7% of all invited schools) with 1014 Year 5 and 6 students (usually between 9–11 years old). A total of 465 children took part in PEDALS of which 398 (85.6%) had complete data on socio-demographics, body composition, physical activity, dietary patterns, CRF, and muscular fitness (Figure 1).

Children’s mean ± SD age was 9.7 ± 0.7 years, ranging from 9 to 11 years old. The majority (82.7%) of participants were New Zealand European and a large number of participants (82.4%) belonged to families with middle or high socio-economic status, based on NZDep13 standards (NZDep13, score 1–7). Three quarters of participants (75.8%) were classified as having a normal BMI (data not shown).

### 3.2. Dietary Intake and Identification of Dietary Patterns

There was a total of 35 missing FFQ data points from 32 participants (0.3% of the entire PEDALS-FFQ dataset). The majority of children had fruit (65%), vegetables (54.8%), and standard milk (~3.3% total fat) (41.7%) every day. Daily consumption of salty snacks was low: 8% for potato chips, potato snacks, and corn chips, and 6% for hot chips, wedges, and French fries. Similarly, only 10% of children consumed sweet snacks such as lollies (i.e., confectioneries or candies), snack bars, chocolate, and chocolate bars on a daily basis (data not shown).

Two dietary patterns were identified, explaining 37.9% of the total variation in diet; “Snacks” and “Fruit and Vegetables”. The “Snacks” pattern was highly positively loaded for salty/sweet snacks, sweet baked goods (e.g., biscuits and fruit pies), lollies, fruit juice, diet/standard fizzy drinks, ice cream, white bread, and pasta. The “Fruit and Vegetables” pattern was highly positively loaded for vegetables (including potato), fruit, milk, yoghurt, cheese, brown bread, breakfast cereal, and meat.

### 3.3. Bivariable Predictors of CRF and Muscular Fitness

As shown in Table 1, being older and a girl was significantly associated with lower $\dot{V}$O_2max_. In addition, overweight-obese participants had lower $\dot{V}$O_2max_ than their normal weight counterparts (β = −3.22, 95% CI = −4.45, −2.78 mL·kg^−1^·min^−1^). In the bivariable model assessing the associations between $\dot{V}$O_2max_ and measures of body composition all were significantly inversely associated with relative $\dot{V}$O_2max_. There was no significant relationship between $\dot{V}$O_2max_ and moderate-vigorous physical activity or dietary pattern scores. 

In the bivariable model (Table 1), assessing the statistical predictors of muscular fitness, being older and a boy was positively associated with muscular fitness of the dominant hand. Overweight-obese participants had 1.35 kg (95% CI = 0.59, 2.10 kg) higher muscular fitness of the dominant hand than their underweight-normal weight counterparts. As shown in Table 1, both FMI and FFMI were positively associated with muscular fitness of the dominant hand. The “Fruit and Vegetables” pattern was also positively significantly associated with muscular fitness of the dominant hand. Similar results were obtained regarding muscular fitness of the non-dominant hand (data not shown).

### 3.4. Independent Relationship Between Dietary Pattern Scores with CRF and Muscular Fitness

As shown in Table 2, there were no significant associations between the dietary pattern scores and relative $\dot{V}$O_2max_ in either the age-and-sex adjusted or fully adjusted models. There was a significant inverse association between the “Snacks” score and muscular fitness of the dominant hand in the fully adjusted model only. The “Fruit and Vegetables” score was positively associated with muscular fitness of the dominant hand in both the age-and-sex adjusted (Model 1) and fully adjusted model (Model 2).

No sex interaction was found in any of the models. However, a weight status interaction was found in Model 2 assessing the relationship between the “Fruit and Vegetables” score and CRF (interaction term *p* = 0.014). To clarify the potential moderation effect of weight status, the fully adjusted regression models were run separately for each of the weight status categories (underweight-normal and overweight-obese). Stratification showed that greater consumption of a “Fruit and Vegetables”-type diet had weak, non-significant relationships with CRF, that were positive in the “underweight-normal weight” group (β = 0.28, 95% CI = −0.21, 0.76 mL·kg^−1^·min^−1^) and negative in the “overweight-obese” group (β = −0.44, 95% CI = −1.29, 0.41 mL·kg^−1^·min^−1^).

## 4. Discussion

This study investigated the associations between dietary pattern scores and both CRF and muscular fitness in 9–11-year-old children. The healthier dietary pattern “Fruit and Vegetables”, was characterized by higher loadings for fruit, vegetables and milk, among other foods. Someone with a high score for this pattern is likely to have high intakes of anti-oxidants, which can impact CRF positively [12] and calcium, which plays a vital role in bone health [37,38]. We found statistically significant but small relationships between dietary pattern scores and muscular fitness but not CRF.

### 4.1. Relationships between Dietary Patterns and CRF

There were no significant relationships between dietary pattern scores and CRF in this study. In contrast to our findings, a “Fruit and Vegetables” pattern was positively associated with CRF in 14–18 year-old New Zealand adolescents [10]. Furthermore, adherence to the Mediterranean diet was positively associated with CRF in 11–12 [8] and 12–16 year-old Spanish adolescents [9]. The age difference of the participants might explain the observed contradiction. As participants in our study were younger, there are two potential reasons that may influence our results; (1) the lack of hormone changes associated with puberty and (2) parental influence over children’s diet and other lifestyle factors. Fitness has been found to be influenced by endocrinological changes during puberty, where sex hormones (testosterone in boys, estrogen in girls) play a key role in fitness by affecting heart size and its function [13]. Although CRF is partly genetically determined, evidence has shown that it is also associated with healthy lifestyle factors [39,40]. However, we did not observe significant associations between lifestyle factors (i.e., diet) and CRF in our cohort. Parents may have a greater influence over children’s behavior compared to participants in previously reported studies [9,10]. Therefore, younger children generally might be at lower risk of unhealthy behaviors than adolescents who have more autonomy over their behavior. Future research could focus on investigating strategies to help children maintain healthy behaviors while transitioning into adolescence.

In addition, in a study of 10 year-old children in Chile a positive association has been reported between the adherence to the Mediterranean diet and CRF [6], which is in contrast with our findings. The authors suggested that the observed diet-fitness association in Chilean children could be related to other factors such as high physical activity levels, which might have had a positive impact on their CRF. The majority (79.9%) of children in our study met the physical activity guidelines of at least 60 min of moderate or vigorous physical activity per day [41]. However, we did not find a significant association between physical activity and CRF. The majority (99%) of our children had healthy fitness levels, based on FITNESSGRAM standards, which might explain this lack of association. Further research is needed to investigate the diet-fitness relationship in children with lower fitness levels. This could provide further insight on the possible relationship between health-related behaviors such as CRF and diet at this early stage of life. This information could then be used to inform health professionals in planning appropriate nutrition intervention strategies for children with low fitness levels.

Although there were no significant associations between dietary patterns and CRF, our findings showed an inverse relationship between adiposity (FMI) and CRF. The adiposity-CRF relationship has been well documented in other studies as well [6,42,43]. Excess body fat may cause early fatigue and performance difficulties in cardiorespiratory fitness activities [44], which might explain the inverse relationship between adiposity and CRF. Our finding suggests that healthy weight status with lower levels of fat mass is beneficially associated with CRF, as excess body fat may cause poorer performance in 20msrt.

### 4.2. Relationships between Dietary Patterns and Muscular Fitness

The current study found a statistically significant inverse association between the “Snacks” score and muscular fitness, while the “Fruit and Vegetables” score was positively associated with muscular fitness. Although statistically significant, the regression coefficients were small, explaining only a small fraction of the standard deviation (SD) of the muscular fitness for a relatively substantial difference in dietary pattern scores (1SD). Thus, it is unlikely that a clinically meaningful association between the dietary pattern scores and muscular fitness exists. To date, only a limited number of studies have investigated the relationship between muscular fitness and dietary patterns [6,9]. Grao-Cruces and colleagues did not find a significant relationship between adherence to the Mediterranean diet and muscular fitness in 12–16 year-old Spanish adolescents [9]. During puberty, sex hormones (i.e., testosterone and estrogen) promote the secretion of growth hormone and insulin-like growth factor 1 (*IGF*-1), which have an important role in protein metabolism and increased muscular fitness [13]. However, given the age of our participants, sex hormones are unlikely to have played an important role in relation to children’s muscular fitness.

In contrast to our findings, Muros et al. [6] found a positive association between diet (adherence to the Mediterranean diet) and muscular fitness (handgrip strength) in 10 year-old Chilean children. Given the importance of muscular fitness as a health indicator in childhood [5] and the positive relationship between diet and muscular fitness, authors have suggested promoting healthier dietary patterns from early stages of life. It should be noted that the majority of children in our study had healthy eating habits (i.e., a high consumption frequency of healthy foods such as fruit, vegetables, and dairy products) and belonged to families with middle/high socio-economic status (82%). Given that high socio-economic status is positively associated with healthier dietary habits [45], future research in this area could focus on children from families with low socio-economic status to investigate possible diet-muscular fitness association. This could help to identify key populations that would benefit the most from nutrition promotion programs. It seems other factors such as sex and body composition (FFMI) are more important with regards to muscular fitness than food choices in our cohort.

### 4.3. Strengths and Limitations

This study is the first to investigate associations between dietary patterns and physical fitness in children. We had a sample size that was suitable for assessing diet-fitness associations, with the ability to adjust for various potential confounders. The current study was conducted in Dunedin city, which has a less diverse population than some other areas in New Zealand and our findings may not be representative of all 9–11-year-old New Zealand children. However, the PEDALS population closely resembles the population in the city of Dunedin, a University city which is one of the major population centers in New Zealand.

We used the PEDALS-FFQ as a self-reported non-quantitative (without portion sizes) tool to collect dietary data. It is acknowledged that self-reported dietary intake has associated measurement errors, for example children may not report foods within composite dishes such as vegetables in noodles/stir fries. However, a key strength of the PEDALS-FFQ was its validity and reproducibility within the same population [20], which allowed us to accurately assess children’s food consumption frequency. Furthermore, the non-quantitative nature of the PEDALS-FFQ was aimed at minimizing difficulties associated with portion size estimation as children may have limitations in describing and quantifying portion sizes due to their cognitive characteristics [46]. By using the non-quantitative FFQ, we were unable to judge whether the frequency or amount of food consumed plays a more important role in the diet-fitness relationship, if there was any. In addition, we used a dietary pattern approach rather than single nutrients/foods to examine the diet-fitness relationship, which considers the possible synergistic or inhibitory interactions among nutrients in the diet and thus provides information that is more realistic in terms of what people actually eat in combination, compared to single foods or nutrients.

For use within the context of epidemiological studies, a test should be easy to conduct, time efficient, valid and reproducible. Our study included objective measures of CRF and muscular fitness, of which the validity and reproducibility has been well-documented in children [15,18,19]. We also followed a standardized protocol for measuring CRF and muscular fitness to remove any potential impact on children’s performance in the test. However, assessment of $\dot{V}$O_2max_ in 20msrt requires children to run almost to exhaustion to reach their maximal oxygen uptake levels. Since children have lower attention spans for monotonous tasks compared with adults and less motivation and willingness to tolerate the discomfort of the 20msrt, despite verbal encouragement throughout the test, they might lose interest or ability to continue running that may affect their performance. However, this limitation is not unique to our study.

## 5. Conclusions

Our findings show that, in an almost exclusively fit cohort, food choice was not related to fitness. This is in contrast to previous research in other populations. It may be that hormonal changes associated with puberty are driving changes seen in other cohorts. It is also possible that in this age group that parental control over diet and activity is driving cardiovascular positive lifestyle choices in this age group. It is known that as both levels of fitness and healthy food choices decline during the transition into adolescence, future research in this cohort could identify factors that help to preserve these health-related factors during this critical period of growth and development. The majority of children in the current study had healthy levels of CRF (FITNESSGRAM standards) and belonged to families of middle/high socio-economic status. Thus, research is needed to investigate diet-fitness relationship in prepubertal children with lower fitness levels than our population, and children from low socio-economic status, to identify key populations for potential investments in health-promoting behaviors at early stages of life.

## Figures and Tables

**Table 1:** Bivariable predictors of CRF and muscular fitness.

Variable	*n* (%)	$\dot{V}$O_2max_, mL·kg^−1^·min^−1^		Muscular Fitness, kg	
				Dominant Hand	
		Mean ± SD	*p* Value	Mean ± SD	*p* Value
		48.7 ± 4.75	-	15.23 ± 3.29	-
Sex			0.002		<0.001
Boys	198 (49.7)	49.9 ± 5.18		16.0 ± 3.52	
Girls	200 (50.3)	47.6 ± 3.99		14.5 ± 2.88	
Ethnicity			0.521		0.287
Māori	44 (11.1)	48.4 ± 5.67		15.8 ± 3.39	
Non-Māori	354 (88.9)	48.8 ± 4.63		15.2 ± 3.28	
NZDep13			0.058		0.966
Low deprivation	175 (44.0)	49.7 ± 5.11		15.3 ± 3.18	
Middle deprivation	153 (38.4)	48.5 ± 4.33		15.2 ± 3.31	
High deprivation	70 (17.6)	47.0 ± 4.13		15.2 ± 3.57	
BMI, kg·m^−2^			<0.001		0.001
Underweight-normal weight	323 (81.2)	49.5 ± 4.71		15.0 ± 3.19	
Overweight-obese	75 (18.8)	45.7 ± 3.59		16.3 ± 3.54	
		β (95% CI)	*p* Value	β (95% CI)	*p* Value
BMI Z-score		−1.20 (−1.57, −0.83)	<0.001	0.96 (0.76, 1.16)	<0.001
FMI, kg·m^−2^		−0.80 (−1.00, −0.60)	<0.001	0.24 (0.09, 0.39)	0.002
FFMI, kg·m^−2^		−0.47 (−0.80, −0.13)	0.006	1.24 (0.98, 1.50)	<0.001
Moderate-vigorous physical activity, h		0.02 (−0.07, 0.11)	0.605	0.08 (−0.00, 0.17)	0.062
Snacks score		−0.22 (−0.54, 0.10)	0.179	−0.02 (−0.36, 0.32)	0.897
Fruit and Vegetables score		−0.04 (−0.41, 0.34)	0.850	0.65 (0.30, 1.00)	<0.001
Age, year		−0.91 (−1.56, −0.27)	0.005	1.35 (0.89, 1.81)	<0.001

β represents the mean difference in $\dot{V}$O_2max_ (mL·kg^−1^·min^−1^) or muscular fitness (kg) associated with a unit higher in BMI Z-score, measures of body composition, moderate-vigorous physical activity, dietary pattern scores, and age. n represents number of participants. CRF, cardiorespiratory fitness; $\dot{V}$O_2max_, maximal oxygen uptake; NZDep13, New Zealand deprivation index; BMI, body mass index; FMI, fat mass index; FFMI, fat-free mass index.

**Table 2:** Relationships between dietary pattern scores and CRF and muscular fitness.

Variable	Snacks			Fruit and Vegetables		
	β	(95% CI)	*p* Value	β	(95% CI)	*p* Value
$\dot{V}$O_2max_, mL·kg^−1^·min^−1^						
Model 1	−0.33	−0.69, 0.04	0.077	0.02	−0.41, 0.46	0.913
Model 2$\dot{V}$O_2max_	−0.22	−0.49, 0.04	0.099	0.15	−0.23, 0.53	0.430
Muscular fitness (Dominant hand), kg						
Model 1	−0.17	−0.45, 0.11	0.238	0.53	0.19, 0.88	0.003
Model 2 Muscular fitness	−0.24	−0.41, −0.06	0.007	0.31	0.02, 0.59	0.037

Model 1: adjusted for age and sex. Model 2 $\dot{V}$O_2max_: Model 1 + ethnicity, NZDep13, FFMI, FMI, height, and moderate-vigorous physical activity. Model 2 Muscular fitness: Model 1 + ethnicity, NZDep13, FFMI, height, and moderate-vigorous physical activity. β represents the mean difference in $\dot{V}$O_2max_ (mL·kg^−1^·min^−1^) or muscular fitness (kg) associated with a unit higher in standardized dietary pattern score. CRF, cardiorespiratory fitness; $\dot{V}$O_2max_, maximal oxygen uptake; FFMI, fat-free mass index; FMI, fat mass index; NZDep13, New Zealand deprivation index 2013.
